# Advancing Health Services Collaborative and Partnership Research Comment on "Experience of Health Leadership in Partnering with University-Based Researchers in Canada – A Call to ‘Re-imagine’ Research"

**DOI:** 10.15171/ijhpm.2020.16

**Published:** 2020-02-15

**Authors:** Monica E. Nyström, Helena Strehlenert

**Affiliations:** ^1^Department of Learning, Informatics, Management and Ethics (LIME), Medical Management Centre, SOLIID, Karolinska Institutet, Stockholm, Sweden.; ^2^Department of Epidemiology and Global Health, Umeå University, Umeå, Sweden.; ^3^Stockholm Gerontology Research Centre, Stockholm, Sweden.

**Keywords:** Research Partnerships, Collaborative Research, Integrated Knowledge Translation, Health Services Research, Sweden

## Abstract

Bowen et al highlight the trend towards partnership research to address the complex challenges currently facing healthcare systems and organizations world-wide. They focus on important strategic actors in partner organizations and their experiences, views and advice for sustainable collaboration, within a Canadian context. The authors call for a multi-system change to provide better conditions for research partnerships. They highlight needs to re-imagine research, to move beyond an ‘acute care’ and clinical focus in research, to re-think research funding, and to improve the academic preparation for research partnerships. In this commentary we provide input to the discussion on practical guidance for those involved in research partnerships based on our partnership experiences from ten research projects conducted within the Swedish healthcare system since 2007. We also highlight areas that need attention in future research in order to learn from approaches used for collaborative and partnership research.

## Introduction

How to improve the use of research in healthcare policy and practice is a recognized, world-wide concern. To enhance a faster and more systematic use of knowledge and provide more useful research is considered important, and the demands for more interdisciplinary and collaborative research approaches has increased.^1-6^ Collaboration between researchers and knowledge users is seen to positively affect research utilization.^7^ Within health services and health systems research co-production is seen as important. However, knowledge about research partnerships that address health systems design and organization, or the actual experiences of such processes is still sparse.^8^

Bowen and colleagues^9^ highlight this trend towards partnership research to address complex challenges in healthcare. They focus on the views of actors on strategic or higher management levels in partner organizations, thereby complementing previous research, which is mostly based on assumptions of researcher-driven initiatives and newly established collaborations.^10^ Lack of more detailed practical guidance is also a concern for those interested in embarking on the partnership journey. Based on experiences from Sweden, we attempt to provide additional input on how to enhance research partnerships, related to Bowen and colleagues’ call for multi-system actions.

## Strategic Level Partners’ Views and Experiences in Canada

It is assumed that research partnerships can enable and enhance the use of research and increase the relevance of research to end-users. However, the strategic actors in Bowen and colleagues’ study provide a fragmented description of research partnerships and a narrow view of research. Research is perceived to be of limited use, especially in organizational decision-making, and research partnerships are given low priority. They describe a lack of shared understanding and tension regarding concepts of research and quality improvement. In addition, quality improvement, evaluation and research are perceived as separate activities performed by different actors. Moreover, ‘knowledge translation’ is perceived mainly as the communication of research findings.

Challenges highlighted in Bowen et al relate to restructuring of health systems and ongoing organizational changes. Differing time perspectives between the partner organizations’ and the researchers’ contexts are well-known causes for tensions. Challenges also concern the lack of appropriate organizational infrastructure, the cost of research to the partner organization and stress among staff in liaison roles. Furthermore, there are challenges related to the mismatch of researcher and health system interests and needs, researchers’ lack of understanding of the health system context and collaborative skills. Issues mentioned relate to academic responsiveness, readiness of researchers to work in partnership and systemic issues such as funder requirements (eg, timelines), lack of government support and failure to provide linkages between regions.

To improve research partnerships Bowen et al recommend actions in partner organizations aimed at increasing leaders’ interest in research, enhancing support from strategic level decision-makers, and increasing the amount of staff with formal research experience. Recommendations also involve changes within academia and among funders eg, developing infrastructures to support partnerships. They highlight the need for clear and well-established organizational processes and long-term research-partner relationships.

Based on their findings, Bowen et al call for a multi-system change to provide better conditions for research partnerships. They highlight needs to ‘re-imagine research,’ to move beyond an ‘acute care’ and clinical focus in research, to re-think research funding, and to improve the academic preparation for health services research partnerships. They conclude that new ways of supporting research partnerships in the field of health services/health systems research is required if research is to enhance the dealing with the complex challenges at hand.

## Enhancing Partnership Research in Practice – Experiences From Sweden

In this section we present input to the discussion on practical guidance for research partnerships based on our experiences from ten collaborative and action-oriented research projects in Sweden (see Table 1). Most of these projects have focused on how to organize and facilitate improvement, development and learning in health and social care organizations. We have collaborated with a variety of actors on different levels of the healthcare system. In nation-wide projects (no. 1-5, see Table 1), collaboration has involved national intervention teams and regional actors from Sweden’s 21 healthcare regions. In regional projects, we have collaborated with regional managers, development units and intervention teams (no. 6-10).

**Table 1:** Description of the 10 Swedish Collaborative Research Projects (2007-2022) That the Learnings Are Based on

**Time Period**	**Projects**	**Focus of the Research Project**	**Levels Involved**	**Research Collaboration With**	**Example of Project Publications**
2009-2011	1) Safe delivery	Patient safety in delivery care. Interventions and project initiated by three professional organizations and a national insurance agency. Effects of national and regional interventions	National, Regional	Patient insurance LÖF; Swedish Society of Obstetrics and Gynecology; the Neonatal Section of the Pediatric Society; Swedish Association of Midwives	^11^
2009-2013	2) National guidelines for health promotion – the challenging journey from evidence to clinical practice	Guideline development and implementation, evidence-based practice. Contextual influence on implementation, organizational readiness for change	National, Regional	Swedish National board for Health and Welfare; the public health section in a healthcare region	^12,13^
2012-2014	3) Better life for ill older people	Improved care of older people in 5 target areas. Policy implementation, national, regional and local change and implementation strategies, change processes, effects of strategies and interventions	National, Regional	SALAR, regional development coaches, joint leadership teams healthcare regions/municipalities	^14,15^
2012-2016	4) National initiative to develop the structure and use of NQRs	Increased use of NQRs for quality improvement, research and patient interaction. National and regional implementation and support strategies	National, Regional	SALAR (national office for NQRs); six regional NQR centers, NQR registry holders	^16^
2017-2022	5) National initiative for improving delivery care and women’s health	National, regional and local support strategies for implementation and change, change processes, effects of support strategies and interventions	National, Regional, Local	SALAR; regional program teams in all of Sweden’s 21 regions, local initiatives and their effects in specific intervention areas	Ongoing
2007-2011	6) Sustainability in innovations and organizational learning in healthcare: 1) Building a learning healthcare organization	A healthcare region’s attempt to build a learning organization. Organizational learning and change, organizational support structures, change process	Regional	Regional development unit; strategic level management, unit managers in a region’s specialized hospital care	^17,18^
2007-2011	7) Sustainability in innovations and organizational learning in healthcare: 2) A health promotion program for children 0-18 years	Primary prevention program development, interventions, implementation support, change strategies, effects of interventions, sustainability	Regional	A healthcare region’s public health section and development unit. Child healthcare and primary care	^19^
2009-2013	8) Future welfare services	Development and test of a multi-level strategy for building competence and capacity in organizational change and learning, development and test of methods and tools enhancing change and learning	Regional	A regional R&D unit in a Swedish county; two of 9 municipalities in the region and their divisions of elderly care and care of people with disabilities: division managers, quality developers and unit managers	^20^
2010-2013	9) Development of regional strategies for structured support to parents	Parental support programs, ICDP (a parental program) implementation strategies and support	Regional	The Public health section and the child healthcare in a healthcare region	^21^
2016-2020	10) New forms for supporting innovative development in large and complex healthcare organizations	Comparing regional organization of and strategies for different types of development. Organizational development of competence and capacity in leading and supporting development	Regional, Local	Regional management and the development units in two healthcare regions	Ongoing

Abbreviates: NQRs, national quality registers; LÖF: Landstingens Ömsesidiga Försäkringsbolag; SALAR, Swedish Association of Local Authorities and Regions; ICDP, the International Child Development Programme.

The main learnings from these research collaborations are summarized under four headings in Table 2. Firstly, to perceive and discuss *research partnerships as a journey of mutual learning* has been important for gaining trust, developing mutual understanding and enhancing development of competence and capacity – on behalf of both parties. However, diverse views on knowledge and on the researcher-partner relationship has sometimes been a challenge. Differences in assumptions regarding knowledge, knowledge production and learning can cause tensions.^4,19,22,23^ A key to handle this diversity has been an openness for discussion and the process of jointly visualizing the organizational context, system, actors and processes and create shared mental models of what to achieve and how.^20^ A joint purpose of learning, visualization and regular meta-reflections has enabled all involved parties to be more equal, participating with different experiences, knowledge and perspectives. This way training in research collaboration and development can be part of the research process, if recognized as a joint and necessary learning process.

**Table 2:** Learnings From 10 Collaborative Health Services Research Projects in Sweden

**View research partnerships as a journey of mutual learning**
Learning is enhanced by creating and visualizing shared mental models of the research process, the overarching problem, the research questions and the organizational development needs. Learning can be integrated in the research process by involving both senior staff with previous experience of research-partnering and junior staff (eg, PhD students and new employees in the partner organization). A joint learning process in a research partnership can build individual as well as organizational competence and capacity for research, learning and development, both within the partner organization and the academic setting.
**Choose an overarching research question to strengthen the sustainability of the partnership**
Formulate an overarching research question that will not lose relevance during the partnership period (ie, relevant and not too narrow). The overarching research question should allow for adaptation of the more specific research questions or hypotheses, and for the development of new questions, to mirror changes in research knowledge, within the partner organization, and in society at large.
**Communicate and use rapid feedback-discussion-learning loops**
To continuously gather information to develop an understanding of the different actors’ situation and contexts will aid communication and learning for all involved. Communication during a research partnership requires the consideration from all partners. Consider different communication arenas, and when and for what content and actors they are appropriate. Adjust the content, message, pace of communication, and the use of technical terms and jargon in relation to different purposes and types of actors. Develop the ability to adapt and flexibly adjust the communication during a communication or a meeting if needed. Adapt the ‘work pace’ for different purposes – aim for quick and actionable results to enable fast feedback and discussions with partners and allow for more rigorous (and time-consuming) analyses to produce scientific publications.
**Build and nourish long-term research-partner relationships**
Relationships, and the quality of relationships, between individuals as well as groups are very important to foster partnerships. With few persons as key connections within a partner organization or a researcher context, partnerships become more vulnerable. Long-term contracts including frameworks for collaboration, which also allows for flexibility if the conditions changes, can protect the partnership project in a dynamic organizational context. Positive and trustful relationships are the glue in partnerships, but they need time to develop.

Secondly, to *choose an overarching research question to strengthen the sustainability of the partnership* has been important for securing the perceived relevance among researchers and partners over time, and for the longevity of the partnership. A broad, overarching research question, eg, regarding strategies to support change on multiple levels within a national initiative, leaves some leeway for future changes in interventions or deeper investigations of issues that turn out to be of special relevance to partners, eg, local strategies for involving pregnant immigrant women in the planning of child delivery (project no. 5). Other examples of new research questions that have evolved during projects to capture emerging phenomena, or new interventions, concern eg, hybrid national-regional support structures^16^ and monitoring and follow-up strategies.^17^ However, this requires partners,’ researchers’ and funders’ trust in the researchers’ abilities to be flexible and deliver results. Compared to a more traditional approach where research questions are generated and “owned” by researchers, this way of working has offered flexibility and greater opportunities to respond to different interests.

Thirdly, developing ways *to communicate and to use rapid feedback-discussion-learning loops* with involved actors has been key to mutual learning and use of knowledge in ongoing development processes. This has helped us to adapt research activities to current organizational situations. Stakeholder analysis is a vital part of project management in R&D ventures.^24^ Boaz et al^25^ have provided guiding principles to enhance stakeholder engagement in research that we have found helpful when building relationships. Organizational partners can be heterogeneous, representing several units, functions and/or professions on various system levels. Different modes of communications may be needed to optimize collaboration with different types of stakeholders. For example, we have used different media for communication, played different roles and used different types, length and content in meetings with various actors. To enhance participation, we have often used distance-technology and existing forums in the partner organizations. However, adapting the communication to the current situation in the partner organization is a shared responsibility that is facilitated by longer relationships. Rapid feedback-loops require quick assessments of data and observations so that results can be used both in the partner organization, and in the research project. Finding appropriate approaches to communication and renewing them when needed has been a vital and continuous process in all our projects.

Finally, research partnerships can be enhanced by a mutual interest in *building and nourishing long-term research-partner relationships*. Relationships are less vulnerable if more persons are involved as individuals may leave organizations for different reasons. Several of our partnerships have lasted for many years, the longest ongoing partnership started in 2007 (no. 6,7,9,10). Besides good social relations a sustained partnership needs funding and support from decision-makers. Our collaboration with regions has depended on the ability of the researchers to apply for, and receive, external research grants. Our research collaborations with national actors have mostly been financed by the Ministry of Health and Social Affairs, via Swedish Association of Local Authorities and Regions (SALAR), based on recommendations and/or previous partnership experiences (projects 3-5). In some projects (no 1, 3-5,8,10), partnership contracts have helped to sustain collaboration through turbulent times. In Sweden, some research funders even require such contracts, and provide resources to partner organizations if they meet certain expectations, eg, allocate time and resources, initiate development initiatives (no. 8,10). In projects no 3-5 contracts have included descriptions of the partnership.

## Suggested Future Research on Research Partnerships

For *future research* there is a need to clarify the types of infrastructures and strategies used for enhancing research partnerships and assumptions behind them. It is important to investigate how strategies relate to various contexts, to research questions, interventions, and to the funding involved. Moreover, there is a need to evaluate these strategies’ effects on the partnership process and on knowledge utilization over time. We agree with Bowen et al that the potential benefits and disadvantages of ways to organize collaboration between partner organizations and researchers requires further attention. This includes studies on embedded roles within the organizational system, network structures, and features of recent collaborative and partnership research initiatives world-wide.^26,27^ A recent comparative study in the United States, Brazil, and Spain highlighted “the nuanced nature of involving practitioners based on the context, cultural norms around practitioner roles, available funding for training and compensation, and accepted practices for researchers”^28^ (p. 6). The authors call for cultural humility, a negotiation of interests and pursuits between researchers and practitioners, and mutual support to overcome differences and achieve consensus. More elaborated knowledge on the influences from the practitioners,’ researchers,’ and funders’ contexts, and the support structures they provide on all levels, is needed. Research partnerships also has the potential to combine the different missions of universities, ie, to provide education and learning, to conduct research and create new knowledge, and to contribute to the welfare of the society. These are the missions for Swedish universities, and partnership research can be one way to fulfill them simultaneously.

The conditions and actual work situation for actors involved in partnership research has not been thoroughly addressed. For example, high pressure and constantly changing work conditions due to health system stress and internal organizational change can jeopardize both the potential results and the partnership itself. The work situation of involved actors, the demands related to the research process, and the political aspects on efficient use of resources and how these factors influence collaboration deserves attention. Studies of complex and dynamic organizational phenomena on multiple system-levels often require interdisciplinary and participatory approaches,^4^ mixed-methods and longitudinal research designs.^29^ This puts special demands on the research team and on the partnerships, as well as on the funding agency. Also, the time it takes to establish well-functioning working relationships within new research groups and partnerships is often underestimated.^9^ Calls have been made for funders to take on a bigger role to facilitate and sustain partnerships in the projects that are awarded grants.^5^ However, there is little research to guide decisions on the various forms of infrastructures to support partnership research.

Finally, future studies on how to support and sustain research partnerships can benefit from knowledge from other research areas, eg, inter-organizational collaboration and social partnerships. The latter are “collectivities of organizations that come together to solve ‘messy problems’ that cannot typically be solved by an organization acting alone”^30^ (p. 21). Factors that influence collaborative advantage, and inertia found in this field, share similarities with obstacles found and advices given regarding research partnerships. In our experience, a well-nurtured research partnership can be a long-term relationship building on previous joint experiences and knowledge combined with the ability to handle the dynamics of external influences together. Altogether, this can result in new knowledge, healthcare development, new research questions, and ideas for future partnership projects.

## Ethical issues

Not applicable.

## Competing interests

Authors declare that they have no competing interests.

## Authors’ contributions

Both authors contributed to the writing of the manuscript and both approved the final manuscript.

## Authors’ affiliations

^1^Department of Learning, Informatics, Management and Ethics (LIME), Medical Management Centre, SOLIID, Karolinska Institutet, Stockholm, Sweden. ^2^Department of Epidemiology and Global Health, Umeå University, Umeå, Sweden. ^3^Stockholm Gerontology Research Centre, Stockholm, Sweden.
